# Cancer Fatalism Among Asian American Adults by Origin Group, 2012–2022

**DOI:** 10.1002/cam4.70738

**Published:** 2025-03-18

**Authors:** Justine Liu, Yenan Zhu, Ryan Suk, Milkie Vu

**Affiliations:** ^1^ Department of Preventive Medicine, Feinberg School of Medicine Northwestern University Chicago Illinois USA; ^2^ Department of Health Services Research, Management and Policy, College of Public Health and Health Professions University of Florida Gainesville Florida USA; ^3^ Nell Hodgson Woodruff School of Nursing Emory University Atlanta Georgia USA; ^4^ Department of Health Policy and Management, Rollins School of Public Health Emory University Atlanta Georgia USA; ^5^ Winship Center for Cancer Health Equity Research Winship Cancer Institute of Emory University Atlanta Georgia USA; ^6^ Robert H. Lurie Comprehensive Cancer Center Northwestern University Chicago Illinois USA; ^7^ Department of Medical Social Sciences Northwestern University Chicago Illinois USA

**Keywords:** Asian Americans, cancer fatalism, data disaggregation, health equity, Health Information Trends Survey

## Abstract

**Backgrounds:**

Cancer fatalism, the belief that cancer is predetermined and unpreventable, is associated with lower uptake of cancer prevention. Little is known about cancer fatalism prevalence within various Asian origin groups.

**Methods:**

We conducted a disaggregated analysis of cancer fatalism among Chinese, Filipino, Indian, Vietnamese, and other Asian respondents using the 2012–2022 Health Information National Trends Survey. Pairwise comparisons were conducted to assess differences between each racial and ethnic group.

**Results:**

Significantly lower proportions of Indian respondents (40.36%) endorsed the statement “It seems like everything causes cancer,” when compared with Vietnamese (74.59%, p = 0.0002) and Filipino (75.18%, p = 0.0009) respondents. Lower proportions of Indian and Chinese respondents endorsed the statement “There's not much you can do to lower your chances of getting cancer” when compared with Vietnamese and Filipino respondents, though these differences were not significant.

**Conclusions:**

Findings highlight the heterogeneity among Asian origin groups and emphasize the importance of disaggregated data collection by origin group, which can inform culturally tailored interventions.

## 1 Introduction

Cancer fatalism is generally conceptualized as the belief that cancer diagnosis is uncontrollable and predetermined. It encompasses a perceived inability to prevent or recover from cancer [1, 2, 3], negative emotions regarding cancer outcomes, such as hopelessness, and related feelings [4]. Existing research shows that cancer fatalism is associated with low knowledge about preventative behaviors and greater cancer‐related anxiety [4, 5, 6]. As a result, it negatively impacts cancer prevention behaviors, such as physical activity, healthy diet, not smoking, and cancer screenings [7, 8]. Cancer fatalism has been shown to be higher among socioeconomically disadvantaged and racial and ethnic minoritized groups [3, 4]. Some research indicates that Asian Americans are more likely to view the development of cancer as predetermined or inevitable compared with their counterparts [9, 10], and this fatalistic view often leads to lower cancer screening uptake [11, 12].

There are approximately 22 million Asian Americans, and this population is projected to reach 46 million by 2060 [13, 14]. Asian Americans come from 30+ countries and speak 100+ languages and dialects with unique histories, socioeconomic profiles, and religious and cultural beliefs [15, 16, 17]. Yet, research on cancer outcomes and behaviors of Asian Americans frequently combines all Asian origin groups (e.g., Japanese, Korean, Vietnamese) into a single category [12, 18, 19], which masks potential disparities experienced by the origin group.

While some research has explored cancer fatalism in Asian Americans, they have mostly reported aggregated data for Asian Americans as a whole instead of disaggregating levels of cancer fatalism by origin group [9, 10]. Our study builds upon few existing studies that explore variations in cancer fatalism among distinct Asian origin groups [20]. Our study enriches the existing literature by providing updated recent data and including comparative analyses with Hispanic and non‐Hispanic Black groups, thereby offering a broader perspective on cancer fatalism across diverse populations.

Using the latest data from the Health Information National Trends Survey (HINTS) [21], we conducted a disaggregated analysis of cancer fatalism among Asian origin groups (Chinese, Filipino, Indian, Vietnamese, and other Asian respondents), Hispanic, non‐Hispanic White (NHW), and non‐Hispanic Black (NHB) groups. This study provides insights for the development of tailored strategies for diverse Asian American origin groups that improve understanding about cancer.

## 2 Methods

We analyzed cancer fatalism using seven cycles of HINTS (2012–2022), a nationally representative cross‐sectional survey administered by the National Cancer Institute. The complex survey weights were generated annually to adjust for nonresponse and implemented through a jackknife replication method with 50 replications. For multiple years of data analysis, composite weights were employed. Additional details about HINTS are included in Appendix A. The survey consists of cancer fatalism‐related questions with response options on a Likert scale (1 = Strongly agree to 4 = Strongly disagree). We analyzed two statements: “It seems like everything causes cancer” and “There's not much you can do to lower your chances of cancer.” The primary exposure was racial and ethnic groups: response options in the survey included Chinese, Filipino, Indian, Vietnamese, or of other Asian origins (e.g., Korean, Japanese). NHW, NHB, and Hispanic respondents were included for comparative purposes. Appendix B provides detailed distributions of the sample by cycle and by sociodemographic characteristics (age, sex, and education).

Bivariate analyses were conducted to estimate and compare weighted proportions across racial and ethnic groups. Pairwise comparisons were conducted to assess differences between each racial and ethnic group. All statistical analyses were conducted using PROC SURVEY procedures in SAS software, version 9.4 (SAS Institute), to account for the complex survey design and weights. Statistical significance was set at a Bonferroni‐corrected threshold of *p* < 1.8 ×10^−3^.

## 3 Results

Of a total of 24,869 participants, there were 4181 Hispanic, 15,695 NHW, 3870 NHB respondents, and 1123 Asian respondents (of whom 25.56% were Chinese, 19.41% Filipino, 19.59% Indian, 10.86% Vietnamese, and 24.58% Other Asian).

**FIGURE 1:**
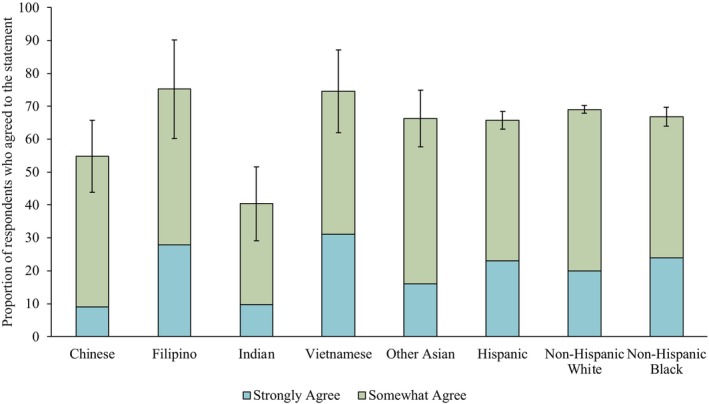
Proportion of respondents indicating they somewhat or strongly agreed to the statement: “It seems like everything causes cancer” by race and ethnicity.

The proportion of Asian respondents who somewhat or strongly agreed with the statement, “It seems like everything causes cancer,” in descending order, were 75.18% for Filipino, 74.59% for Vietnamese, 66.28% for “Other Asian”, 54.77% for Chinese, and 40.36% for Indian respondents (Figure 1). In particular, there was a significantly lower proportion of Indian respondents (40.36%) indicating agreement compared with Filipino (75.18%, *p* = 0.0009), Vietnamese (74.59%, *p* = 0.0002), and “Other Asian” (66.28%, *p* = 0.0005) respondents. Moreover, there was a significantly lower proportion of Indian respondents indicating agreement compared with Hispanic (65.81%, *p* = 0.0003), NHW (69.01%, *p* < 0.0001), and NHB (66.91% [64.04%–69.78%], *p* = 0.0002) respondents. While there was relative heterogeneity in the responses by Asian origin group, the responses among the other racial and ethnic groups appeared to be more similarly distributed. There were no significant differences between proportions of Hispanic, NHW, and NHB respondents who indicated agreement.

We further investigated possible differences in the proportions of respondents who indicated that they strongly agreed with this statement by race and ethnicity (Appendix B). Among Asian participants, there was a significantly lower proportion of Indian (9.76%) respondents indicating strong agreement compared with Filipino (27.81%, *p* = 0.0002) and Vietnamese (31.10%, *p* = 0.0006) respondents. There were no significant differences in the responses of Hispanic (23.04%), NHW (19.98%), and NHB (23.97%) respondents. A significantly lower proportion of Indian and Chinese respondents indicated strong agreement compared with Hispanic, NHW, and NHB respondents (all *p* values < 0.0018).

**FIGURE 2:**
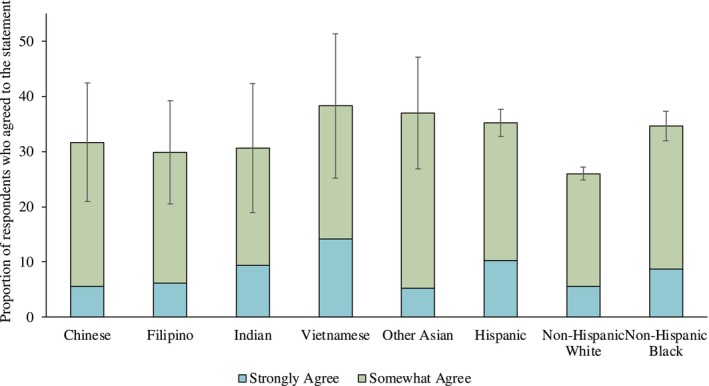
Proportion of respondents indicating they somewhat or strongly agreed to the statement: “There's not much you can do to lower your chances of cancer” by race and ethnicity.

The proportions of Asian respondents that agreed with the statement, “There's not much you can do to lower your chances of getting cancer,” in descending order, were 38.29% for Vietnamese, 36.99% for “Other Asian”, 31.68% for Chinese, 30.63% for Indian, and 29.83% for Filipino respondents (Figure 2). Differences between Asian origin groups did not reach statistical significance when compared with results from Hispanic, NHW, and NHB respondents.

We further investigated possible differences in the proportions of respondents who indicated strong agreement with this statement by race and ethnicity (Appendix B). Among “Other Asian” respondents (5.28%), we observed the lowest proportion of respondents indicating strong agreement, while the highest proportion was seen among Vietnamese respondents (14.19%). Differences across Asian origin groups did not reach statistical significance.

## 4 Discussion

In this study, using nationally representative survey data, we found significant differences in responses to the statement “It seems like everything causes cancer” by Asian origin group. We found that 75.18% of Filipino and 74.59% of Vietnamese respondents indicated agreement, while 40.36% of Indian respondents did so. Moreover, 9.76% of Indian respondents strongly agreed with the statement, while 27.81% of Filipino and 31.10% of Vietnamese respondents did so. Additionally, more Asian respondents in all origin groups agreed with the statement “There's not much you can do to lower your chances of cancer” compared with NHW respondents, even though differences did not reach significance. Findings highlight the need for culturally tailored interventions that consider heterogeneous cancer fatalism levels, as opposed to one‐size‐fits‐all approaches.

Observed differences in cancer fatalism among Asian origin groups may be due to their distinct immigration patterns to the United States and the associated economic motivations and opportunities available to these groups. For example, around half of Indian immigrants obtain US permanent residency through employer sponsorship, while this is less common for Chinese (a fifth), Filipino (one‐in‐eighth), and Vietnamese (1%) immigrants [22]. In contrast to many Asian groups who immigrated for economic and educational reasons, the majority of Vietnamese Americans came to the United States as political refugees [23]. These diverse immigration histories affect educational attainment, English proficiency, and poverty [13], which in turn differentially shape structural barriers to healthcare and, consequently, cancer knowledge and preventative behaviors [24].

Moreover, different Asian origin groups may have varying beliefs about cancer and their acceptance of prevention and treatment methods due to their diverse languages, religions, and cultures [17, 18, 25]. Traditional Chinese medicine views cancer formation as an imbalance of the body's “Yin and Yang,” [26] while Ayurvedic medicine views it as a result of an imbalance between the three doshas (vata, kapha, and pitta—energies believed to govern physiological activity) [27]. While many Chinese Americans (56%) are not religious, the majority of Filipino Americans identify as Christian (75%) [28]. Almost half of Indian Americans identify as Hindu, while more than a third of Vietnamese Americans identify as Buddhist [28]. These religions offer different explanations for cancer and suffering: many Hindus may view suffering as karma, Buddhists as ignorance, and Christians as something to be alleviated by God [29].

Existing literature offers a few examples of interventions to address cancer fatalism [30, 31]. If the belief is rooted in religious faith, emphasizing personal responsibility alongside faith may help [31]. Other strategies can include reducing structural barriers to cancer care through financial assistance programs [32], educational materials to increase health literacy [4], and community health worker‐led programs [33]. Integrating culturally tailored interventions with broader multilevel strategies that reduce structural barriers can effectively target cancer fatalism in diverse populations.

This study has several limitations. There was a relatively small sample size of Asian origin groups in the dataset when compared with Hispanic, NHW, and NHB respondents. A larger sample size may have allowed us to detect more significant differences across groups. Moreover, the dataset did not include variables related to immigration reasons, immigration status (e.g., citizenship or visa status), or acculturation, which could have elucidated additional structural or sociocultural mechanisms underlying cancer fatalism.

## 5 Conclusion

We provide insights into heterogeneous cancer fatalism levels among Asian origin groups. Understanding these differences is critical for designing culturally tailored interventions addressing cancer fatalism and ultimately improving cancer prevention and treatment in diverse Asian American populations.

## Author Contributions

Justine Liu contributed to the writing of the original draft, visualization, and reviewing and editing of the manuscript. Yenan Zhu contributed to the methodology, validation, writing review and editing, software development, formal analysis, and data curation. Ryan Suk contributed to validation and writing review and editing. Milkie Vu, as the corresponding and submitting author, contributed to supervision, writing review and editing, funding acquisition, and project administration.

## Conflicts of Interest

The authors declare no conflicts of interest.

## Data Availability

Data supporting the findings of this study are publicly available in the Health Information National Trends Survey database from the National Cancer Institute.

## Appendix A

This study used data from the US Health Information National Trends Survey (HINTS), administered by the National Cancer Institute (NCI). The survey is designed to assess the American's public's knowledge, attitudes, and behaviors regarding cancer‐related information across a nationally representative sample of civilian non‐institutionalized adults aged 18 years and older. Data were collected through seven survey cycles: 2012 (*n* = 3630), 2013 (*n* = 3185), 2014 (*n* = 3677), 2017 (*n* = 3285), 2018 (*n* = 3504), 2019 (*n* = 5438), and 2022 (*n* = 6252). These cycles were combined into a pooled cross‐sectional dataset for analysis. Respondents were selected using a two‐stage sampling design. First, a stratified sample of residential addresses was drawn from a master address file. Subsequently, one adult was randomly selected from each sampled household. Survey weights were applied to adjust for non‐responses and coverage errors, ensuring representative population estimates. Jackknife replicate weights were employed to provide bias‐corrected variance estimates. Details regarding the survey design and methodology are available elsewhere [21].

Our outcome of interest, cancer fatalism, was measured through responses to two statements: “It seems like everything causes cancer” and “There's not much you can do to lower your chances of cancer”, using a 4‐point Likert scale (strongly agree, agree, disagree, strongly disagree). Responses were initially dichotomized into agree versus disagree categories. Further analyses distinguished between respondents who strongly agreed with the statements.

## Appendix B

Tables B1, B2, B3, B4, B5, B6.

**TABLE B1:** Description of racial and ethnic origin groups in the Health Information National Trends Survey by cycle.

	Asian Indian					Chinese				
	*n*	Weighted *N*	%	95% confidence interval (lower limit)	95% confidence interval (upper limit)	*n*	Weighted *N*	%	95% confidence interval (lower limit)	95% confidence interval (upper limit)
HINTS 4 CYCLE 2	18	1,414,160	7.7	3.48	11.92	28	2,651,873	11.63	(6.14, 17.13)	17.13
HINTS 4 CYCLE 3	17	2,035,439	11.09	4.44	17.73	25	2,773,566	12.17	7.32	17.02
HINTS 4 CYCLE 4	16	2,776,759	15.13	6.57	23.68	38	3,686,131	16.17	10.52	21.82
HINTS 5 CYCLE 1	26	2,485,782	13.54	8.14	18.94	36	3,988,764	17.5	11.24	23.76
HINTS 5 CYCLE 2	29	3,905,740	21.28	9.96	32.6	36	2,664,880	11.69	6.33	17.05
HINTS 5 CYCLE 3	51	3,051,661	16.62	9.71	23.54	52	2,229,302	9.78	6.04	13.52
HINTS 6	63	2,687,761	14.64	9.22	20.06	72	4,800,684	21.06	12.89	29.23
Total	220	18,357,302	100			287	22,795,200	100		

	Filipino					Vietnamese				
	*n*	Weighted *N*	%	95% confidence interval (lower limit)	95% confidence interval (upper limit)	*n*	Weighted *N*	%	95% confidence interval (lower limit)	95% confidence interval (upper limit)
HINTS 4 CYCLE 2	18	3,059,542	19.96	5.48	34.44	13	1,398,809	18.57	5.24	31.89
HINTS 4 CYCLE 3	27	2,367,205	15.45	8.05	22.84	12	668,670	8.87	2.12	15.63
HINTS 4 CYCLE 4	24	2,011,698	13.13	5.65	20.6	13	748,700	9.94	2.78	17.09
HINTS 5 CYCLE 1	26	2,409,339	15.72	7.88	23.56	20	1,162,874	15.43	7.38	23.49
HINTS 5 CYCLE 2	27	1,530,450	9.99	4.82	15.15	17	1,256,606	16.68	4.08	29.27
HINTS 5 CYCLE 3	43	2,374,909	15.5	8.8	22.2	20	1,025,125	13.61	4.44	22.77
HINTS 6	53	1,573,052	10.26	5.32	15.21	27	1,273,746	16.91	6.28	27.53
Total	218	15,326,195	100			122	7,534,530	100		

	Other Asian					Non‐Hispanic White				
	*n*	Weighted *N*	%	95% confidence interval (lower limit)	95% confidence interval (upper limit)	*n*	Weighted *N*	%	95% confidence interval (lower limit)	95% confidence interval (upper limit)
HINTS 4 CYCLE 2	21	1,739,283	9.3	4.57	14.03	2043	146,323,695	14.35	14.14	14.55
HINTS 4 CYCLE 3	33	3,193,995	17.08	10.01	24.16	1584	140,407,137	13.77	13.53	14
HINTS 4 CYCLE 4	32	2,056,927	11	6.59	15.41	1960	145,596,461	14.27	14.08	14.47
HINTS 5 CYCLE 1	30	2,576,727	13.78	6.68	20.89	1868	149,554,884	14.66	14.44	14.89
HINTS 5 CYCLE 2	29	2495,004	13.35	7.28	19.42	1983	149,006,915	14.61	14.38	14.83
HINTS 5 CYCLE 3	58	3,608,375	19.3	12.72	25.89	3054	146,092,785	14.32	14.12	14.53
HINTS 6	73	3,024,681	16.18	10.27	22.09	3203	143,047,138	14.02	13.81	14.24
Total	276	18,694,991	100			15,695	1,020,029,014	100		

	Non‐Hispanic Black					Hispanic				
	*n*	Weighted *N*	%	95% confidence interval (lower limit)	95% confidence interval (upper limit)	*n*	Weighted *N*	%	95% confidence interval (lower limit)	95% confidence interval (upper limit)
HINTS 4 CYCLE 2	496	23,732,225	13.95	13.29	14.62	511	32,609,551	12.91	12.57	13.25
HINTS 4 CYCLE 3	421	21,923,564	12.89	12.03	13.75	511	33,972,178	13.45	13.14	13.76
HINTS 4 CYCLE 4	534	24,388,449	14.34	13.69	14.99	540	34,828,619	13.79	13.51	14.06
HINTS 5 CYCLE 1	409	23,395,382	13.76	12.63	14.88	427	35,858,725	14.2	13.78	14.61
HINTS 5 CYCLE 2	444	24,919,004	14.65	13.97	15.33	461	36,779,202	14.56	14.09	15.03
HINTS 5 CYCLE 3	677	26,039,735	15.31	14.75	15.87	730	38,726,741	15.33	15.09	15.57
HINTS 6	889	25,682,584	15.1	14.59	15.61	1001	39,839,133	15.77	15.41	16.13
Total	3870	170,080,943	100			4181	252,614,148	100		

**TABLE B2:** Description of racial and ethnic origin groups in the Health Information National Trends Survey by sociodemographic characteristics (age, sex, and education).

	Asian Indian					Chinese				
	*n*	Weighted *N*	%	95% confidence interval (lower limit)	95% confidence interval (upper limit)	*n*	Weighted *N*	%	95% confidence interval (lower limit)	95% confidence interval (upper limit)
Age										
18–26	17	3,620,215	19.95	4.80	35.09	31	4,753,441	21.11	10.45	31.78
27–45	104	9,488,287	52.28	39.12	65.45	102	9,778,786	43.44	33.56	53.31
46+	96	5,039,621	27.77	18.42	37.12	150	7,980,983	35.45	27.40	43.50
Sex										
Male	136	12,252,752	67.59	57.69	77.49	135	11,749,152	51.54	42.38	60.71
Female	82	5,874,676	32.41	22.51	42.31	152	11,046,048	48.46	39.29	57.62
Education										
Less than a college degree	47	5,453,520	29.95	17.04	42.85	72	6,660,699	29.22	19.82	38.62
College degree and above	171	12,757,568	70.05	57.15	82.96	215	16,134,500	70.78	61.38	80.18

	Filipino					Vietnamese				
	*n*	Weighted *N*	%	95% confidence interval (lower limit)	95% confidence interval (upper limit)	*n*	Weighted *N*	%	95% confidence interval (lower limit)	95% confidence interval (upper limit)
Age										
18–26	12	4,382,557	28.64	14.11	43.18	7	713,653	9.54	1.16	17.92
27–45	61	4,069,565	26.60	18.10	35.10	35	2,776,523	37.12	21.42	52.82
46+	144	6,847,558	44.76	32.23	57.29	78	3,989,587	53.34	37.88	68.79
Sex										
Male	79	7,545,453	50.14	36.86	63.42	54	4,124,378	54.74	39.81	69.67
Female	136	7,503,468	49.86	36.58	63.14	68	3,410,152	45.26	30.33	60.19
Education										
Less than a college degree	61	7,427,645	48.69	30.66	66.73	68	4,799,784	64.59	52.08	77.10
College degree and above	155	7,826,017	51.31	33.27	69.34	52	2,631,167	35.41	22.90	47.92

	Other Asian					Non‐Hispanic White				
	*n*	Weighted *N*	%	95% confidence interval (lower limit)	95% confidence interval (upper limit)	*n*	Weighted *N*	%	95% confidence interval (lower limit)	95% confidence interval (upper limit)
Age										
18–26	18	2,252,751	12.13	4.68	19.58	598	114,340,713	11.31	10.31	12.30
27–45	105	8,398,813	45.22	35.70	54.75	3358	294,989,359	29.17	28.05	30.29
46+	151	7,919,711	42.64	33.89	51.40	11,596	601,859,100	59.52	58.65	60.39
Sex										
Male	134	9,690,944	51.86	42.73	60.99	6619	497,406,615	49.11	48.76	49.46
Female	141	8,995,656	48.14	39.01	57.27	8964	515,406,006	50.89	50.54	51.24
Education										
Less than a college degree	90	7,142,452	38.25	28.77	47.72	7882	676,783,979	66.61	66.27	66.95
College degree and above	185	11,533,000	61.75	52.28	71.23	7742	339,223,101	33.39	33.05	33.73

	Non‐Hispanic Black					Hispanic				
	*n*	Weighted *N*	%	95% confidence interval (lower limit)	95% confidence interval (upper limit)	*n*	Weighted *N*	%	95% confidence interval (lower limit)	95% confidence interval (upper limit)
Age										
18–26	148	18,647,150	11.15	8.64	13.67	306	43,160,577	17.36	15.09	19.63
27–45	907	58,603,362	35.05	32.33	37.77	1372	97,654,909	39.28	36.79	41.76
46+	2745	89,944,650	53.80	50.96	56.64	2444	107,821,772	43.37	41.00	45.74
Sex										
Male	1194	70,776,433	42.29	39.45	45.12	1635	125,336,752	50.16	48.16	52.16
Female	2625	96,595,351	57.71	54.88	60.55	2495	124,528,424	49.84	47.84	51.84
Education										
Less than a college degree	2479	117,186,903	69.16	67.13	71.18	2897	190,317,285	75.88	74.38	77.37
College degree and above	1371	52,267,166	30.84	28.82	32.87	1246	60,504,103	24.12	22.63	25.62

**TABLE B3:** Distribution of responses to the question of “It seems like everything causes cancer” by racial and ethnic group.

		*n*	Weighted *N*	%	95% confidence interval (lower limit)	95% confidence interval (upper limit)
Asian Indian	Missing data (not ascertained)	3	95,517	0.52	0.00	1.22
	Multiple responses selected in error	—				
	Strongly agree	27	1,791,242	9.76	4.50	15.02
	Somewhat agree	75	5,579,922	30.40	20.59	40.20
	Somewhat disagree	55	6,069,309	33.06	19.12	47.00
	Strongly disagree	60	4,821,312	26.26	15.90	36.63
	Total	220	18,357,302	100.00		
Chinese	Missing data (not ascertained)	1	23,381	0.10	0.00	0.31
	Multiple responses selected in error	—				
	Strongly agree	35	2,057,808	9.03	4.75	13.30
	Somewhat agree	128	10,413,579	45.68	35.20	56.16
	Somewhat disagree	72	6,359,769	27.90	19.84	35.96
	Strongly disagree	51	3,940,663	17.29	7.00	27.57
	Total	287	22,795,200	100.00		
Filipino	Missing data (not ascertained)	4	81,565	0.53	0.00	1.12
	Multiple responses selected in error	—				
	Strongly agree	52	4,262,888	27.81	18.18	37.45
	Somewhat agree	95	7,197,395	46.96	31.14	62.79
	Somewhat disagree	41	2,867,903	18.71	4.42	33.01
	Strongly disagree	26	916,444	5.98	2.45	9.51
	Total	218	15,326,195	100.00		
Vietnamese	Missing data (not ascertained)	7	358,841	4.76	0.67	8.86
	Multiple responses selected in error	—				
	Strongly agree	26	2,343,260	31.10	17.65	44.55
	Somewhat agree	48	3,009,227	39.94	26.16	53.72
	Somewhat disagree	26	952,406	12.64	5.69	19.59
	Strongly disagree	15	870,796	11.56	0.99	22.13
	Total	122	7,534,530	100.00		
Other Asian	Missing data (not ascertained)	2	33,721	0.18	0.00	0.44
	Multiple responses selected in error	—				
	Strongly agree	41	2,996,523	16.03	9.42	22.63
	Somewhat agree	121	9,371,767	50.13	40.23	60.03
	Somewhat disagree	82	4,547,306	24.32	17.07	31.57
	Strongly disagree	30	1,745,674	9.34	4.87	13.81
	Total	276	18,694,991	100.00		
Non‐Hispanic White	Missing data (not ascertained)	173	8,683,168	0.85	0.66	1.04
	Multiple responses selected in error	—				
	Strongly agree	2585	203,788,383	19.98	18.82	21.14
	Somewhat agree	7355	494,134,876	48.44	47.17	49.71
	Somewhat disagree	3505	203,312,002	19.93	18.92	20.95
	Strongly disagree	2077	110,110,586	10.79	10.08	11.51
	Total	15,695	1,020,029,014	100.00		
Non‐Hispanic Black	Missing data (not ascertained)	106	3,667,916	2.16	1.49	2.83
	Multiple responses selected in error	5	276,947	0.16	0.00	0.40
	Strongly agree	968	40,765,452	23.97	21.76	26.18
	Somewhat agree	1552	70,400,418	41.39	38.66	44.12
	Somewhat disagree	670	29,333,934	17.25	15.05	19.44
	Strongly disagree	569	25,636,276	15.07	12.59	17.56
	Total	3870	170,080,943	100.00		
Hispanic	Missing data (not ascertained)	103	4,353,865	1.72	1.25	2.20
	Multiple responses selected in error	7	477,784	0.19	0.00	0.40
	Strongly agree	995	58,200,933	23.04	20.84	25.24
	Somewhat agree	1670	104,869,697	41.51	38.64	44.38
	Somewhat disagree	781	45,361,896	17.96	15.97	19.95
	Strongly disagree	625	39,349,974	15.58	13.48	17.67
	Total	4181	252,614,148	100.00		

**TABLE B4:** Distribution of collapsed responses to the question of “It seems like everything causes cancer” by racial and ethnic group (*collapse “strongly agree” and “somewhat agree” as “agree”; collapse “strongly disagree” and “somewhat disagree” as “disagree”*).

		*n*	Weighted *N*	%	95% confidence interval (lower limit)	95% confidence interval (upper limit)
Asian Indian	Disagree	115	10,890,621	59.64	48.45	70.82
	Agree	102	7,371,164	40.36	29.18	51.55
	Total	217	18,261,785	100.00		
Chinese	Disagree	123	10,300,432	45.23	34.25	56.21
	Agree	163	12,471,387	54.77	43.79	65.75
	Total	286	22,771,818	100.00		
Filipino	Disagree	67	3,784,347	24.82	9.78	39.87
	Agree	147	11,460,284	75.18	60.13	90.22
	Total	214	15,244,631	100.00		
Vietnamese	Disagree	41	1,823,201	25.41	12.85	37.96
	Agree	74	5,352,487	74.59	62.04	87.15
	Total	115	7,175,689	100.00		
Other Asian	Disagree	112	6,292,980	33.72	25.12	42.33
	Agree	162	12,368,290	66.28	57.67	74.88
	Total	274	18,661,270	100.00		
Non‐Hispanic White	Disagree	5582	313,422,588	30.99	29.82	32.16
	Agree	9940	697,923,258	69.01	67.84	70.18
	Total	15,522	1,011,345,846	100.00		
Non‐Hispanic Black	Disagree	1239	54,970,210	33.09	30.22	35.96
	Agree	2520	111,165,870	66.91	64.04	69.78
	Total	3759	166,136,080	100.00		
Hispanic	Disagree	1406	84,711,870	34.19	31.47	36.90
	Agree	2665	163,070,630	65.81	63.10	68.53
	Total	4071	247,782,499	100.00		

**TABLE B5:** Distribution of responses to the question of “There's not much you can do to lower your chances of cancer” by racial and ethnic group.

		*n*	Weighted *N*	%	95% confidence interval (lower limit)	95% confidence interval (upper limit)
Asian Indian	Missing data (not ascertained)	4	154,213	0.84	0.00	1.79
	Multiple responses selected in error	—				
	Strongly agree	18	1,717,700	9.36	0.29	18.43
	Somewhat agree	50	3,858,286	21.02	12.66	29.38
	Somewhat disagree	87	8,424,320	45.89	33.03	58.75
	Strongly disagree	61	4,202,783	22.89	14.96	30.83
	Total	220	18,357,302	100.00		
Chinese	Missing data (not ascertained)	—				
	Multiple responses selected in error	—				
	Strongly agree	19	1272,621	5.58	1.42	9.74
	Somewhat agree	77	5,948,037	26.09	15.45	36.74
	Somewhat disagree	109	9,395,395	41.22	31.08	51.35
	Strongly disagree	82	6,179,147	27.11	18.16	36.06
	Total	287	22,795,200	100.00		
Filipino	Missing data (not ascertained)	7	144,800	0.94	0.10	1.79
	Multiple responses selected in error	—				
	Strongly agree	15	942,536	6.15	2.11	10.19
	Somewhat agree	64	3,586,073	23.40	13.90	32.89
	Somewhat disagree	73	6,204,384	40.48	24.46	56.50
	Strongly disagree	59	4,448,403	29.02	13.81	44.24
	Total	218	15,326,195	100.00		
Vietnamese	Missing data (not ascertained)	7	358,841	4.76	0.67	8.86
	Multiple responses selected in error	—				
	Strongly agree	11	1,069,133	14.19	4.36	24.02
	Somewhat agree	41	1,678,193	22.27	13.23	31.32
	Somewhat disagree	47	3,356,841	44.55	30.35	58.76
	Strongly disagree	16	1,071,521	14.22	3.17	25.28
	Total	122	7,534,530	100.00		
Other Asian	Missing data (not ascertained)	2	59,614	0.32	0.00	0.81
	Multiple responses selected in error	—				
	Strongly agree	11	987,370	5.28	1.14	9.43
	Somewhat agree	71	5,906,773	31.60	21.53	41.66
	Somewhat disagree	127	6,942,856	37.14	28.36	45.92
	Strongly disagree	65	4,798,379	25.67	16.76	34.57
	Total	276	18,694,991	100.00		
Non‐Hispanic White	Missing data (not ascertained)	189	9,138,702	0.90	0.70	1.09
	Multiple responses selected in error	3	321,554	0.03	0.00	0.09
	Strongly agree	779	57,221,212	5.61	4.98	6.24
	Somewhat agree	3034	205,308,320	20.13	19.06	21.19
	Somewhat disagree	6535	442,094,409	43.34	41.95	44.73
	Strongly disagree	5155	305,944,817	29.99	28.77	31.22
	Total	15,695	1,020,029,014	100.00		
Non‐Hispanic Black	Missing data (not ascertained)	138	4,680,603	2.75	1.99	3.51
	Multiple responses selected in error	4	404,683	0.24	0.00	0.53
	Strongly agree	380	14,762,058	8.68	7.32	10.04
	Somewhat agree	892	42,451,979	24.96	22.37	27.55
	Somewhat disagree	1228	51,769,508	30.44	27.97	32.91
	Strongly disagree	1228	56,012,112	32.93	30.04	35.83
	Total	3870	170,080,943	100.00		
Hispanic	Missing data (Not Ascertained)	134	6,284,202	2.49	1.84	3.13
	Multiple responses selected in error	6	306,547	0.12	0.00	0.29
	Strongly agree	451	26,031,377	10.30	8.69	11.92
	Somewhat agree	1039	60,568,758	23.98	21.75	26.20
	Somewhat disagree	1413	91,129,591	36.07	33.42	38.73
	Strongly disagree	1138	68,293,673	27.03	24.63	29.44
	Total	4181	252,614,148	100.00		

**TABLE B6:** Distribution of collapsed responses to the question of “There's not much you can do to lower your chances of cancer” by racial and ethnic group (*collapse “strongly agree” and “somewhat agree” as “agree”; collapse “strongly disagree” and “somewhat disagree” as “disagree”*).

		*n*	Weighted *N*	%	95% confidence interval (lower limit)	95% confidence interval (upper limit)
Asian Indian	Disagree	148	12,627,103	69.37	57.71	81.03
	Agree	68	5,575,985	30.63	18.97	42.29
	Total	216	18,203,088	100.00		
Chinese	Disagree	191	15,574,541	68.32	57.56	79.09
	Agree	96	7,220,658	31.68	20.91	42.44
	Total	287	22,795,200	100.00		
Filipino	Disagree	132	10,652,786	70.17	60.81	79.53
	Agree	79	4,528,609	29.83	20.47	39.19
	Total	211	15,181,395	100.00		
Vietnamese	Disagree	63	4,428,362	61.71	48.62	74.80
	Agree	52	2,747,326	38.29	25.20	51.38
	Total	115	7,175,689	100.00		
Other Asian	Disagree	192	11,741,234	63.01	52.89	73.13
	Agree	82	6,894,143	36.99	26.88	47.11
	Total	274	18,635,377	100.00		
Non‐Hispanic White	Disagree	11,690	748,039,226	74.02	72.86	75.19
	Agree	3813	262,529,532	25.98	24.81	27.15
	Total	15,503	1,010,568,758	100.00		
Non‐Hispanic Black	Disagree	2456	107,781,621	65.32	62.63	68.02
	Agree	1272	57,214,037	34.68	31.98	37.37
	Total	3728	164,995,658	100.00		
Hispanic	Disagree	2551	159,423,264	64.80	62.34	67.26
	Agree	1490	86,600,135	35.20	32.74	37.66
	Total	4041	246,023,399	100.00		
